# Minimal Effects of Age and Exposure to a Noisy Environment on Hearing in Alpha9 Nicotinic Receptor Knockout Mice

**DOI:** 10.3389/fnins.2017.00304

**Published:** 2017-06-02

**Authors:** Amanda M. Lauer

**Affiliations:** Department of Otolaryngology-HNS, Center for Hearing and Balance, Johns Hopkins University School of MedicineBaltimore, MD, United States

**Keywords:** olivocochlear, auditory efferent, age, hearing loss, alpha9 nicotinic acetylcholine receptor, noise

## Abstract

Studies have suggested a role of weakened medial olivocochlear (OC) efferent feedback in accelerated hearing loss and increased susceptibility to noise. The present study investigated the progression of hearing loss with age and exposure to a noisy environment in medial OC-deficient mice. Alpha9 nicotinic acetylcholine receptor knockout (α9KO) and wild types were screened for hearing loss using auditory brainstem responses. α9KO mice housed in a quiet environment did not show increased hearing loss compared to wild types in young adulthood and middle age. Challenging the medial OC system by housing in a noisy environment did not increase hearing loss in α9KO mice compared to wild types. ABR wave 1 amplitudes also did not show differences between α9KO mice and wild types. These data suggest that deficient medial OC feedback does not result in early onset of hearing loss.

## Introduction

Regulation of auditory afferent input to the brain occurs via an efferent pathway termed the olivocochlear (OC) system. Medial OC (MOC) efferents decrease activity in the ear and indirectly affect the auditory input from the cochlea to the brain by reducing the gain produced by outer hair cells via an acetylcholine-triggered mechanism (Fuchs, 2014; Guinan, 2014). Lateral OC efferents (LOC) can decrease or increase auditory nerve activity by direct action on auditory nerve fibers through a suite of different neurotransmitters and neuropeptides (Darrow et al., 2006; Le Prell et al., 2014). The OC system has been implicated in hearing in noise (Kawase et al., 1993; Giraud et al., 1995; Hienz et al., 1998; de Boer and Thornton, 2008), protection from loud noise exposure (Rajan, 1988; Maison and Liberman, 2000; Darrow et al., 2007; Taranda et al., 2009; Fuente, 2015), sound localization (May et al., 2004; Irving et al., 2011), and selective attention (Scharf et al., 1997; Zeng et al., 2000; Terreros et al., 2016).

While OC regulation of afferent activity is known to contribute to developmental refinement of auditory circuits (Clause et al., 2011; Johnson et al., 2013; Hickman et al., 2015), the impact of OC regulation of afferent activity over the course of adult aging is not as well-understood. Studies in humans and animals using contralateral noise suppression of otoacoustic emissions as a measure of MOC strength have suggested that weakened MOC activity contributes to age-related hearing loss (Parthasarathy, 2001; Kim et al., 2002; Jacobson et al., 2003; Varghese et al., 2005; Zhu et al., 2007). Presumably, weakened MOC strength leaves the cochlea more vulnerable to damage to environmental sounds accrued over a lifetime, resulting in earlier onset or more severe age-related hearing loss. However, other studies indicate that weakened MOC strength in aging subjects may itself be due to hearing loss (Castor et al., 1994; Tadros et al., 2005; Keppler et al., 2010; Konomi et al., 2014), presumably because of reduced afferent input driving the MOC neurons or damage to outer hair cells that limits MOC functionality. Other studies have not found evidence of weakened MOC strength in older adults, and MOC activation was even enhanced under certain conditions (Quaranta et al., 2001; Abdala et al., 2014).

The mixed results from contralateral suppression studies in human listeners highlight the difficulties in determining which comes first: weakening of the OC pathways with age or age-related hearing loss. Direct manipulation of OC neurons in animal models has the potential to provide more definitive information. Lesions of the OC neurons triggers accelerated loss of ribbon synapses and auditory brainstem response (ABR) threshold shifts with age and increase susceptibility to damage from chronic noise exposure (Maison et al., 2013; Liberman et al., 2014). Loss of MOC neurons induced by sectioning the crossed OC bundle primarily affects lower to mid frequency regions of the cochlea, whereas damaging LOC neurons with a neurotoxin primarily affects higher frequency regions (Liberman et al., 2014). These findings demonstrate a role for OC protection of hearing with age, but nonspecific effects may complicate the interpretation of lesion studies if other neurons are affected by the surgical procedures or the lesions are incomplete. An alternative approach is to measure hearing loss in genetically modified mouse strains with abnormal OC strength.

Mice missing the alpha9 nicotinic acetylcholine receptor subunit in MOC synapses (α9KO) lack efferent cochlear suppression of distortion product otoacoustic emissions and compound action potentials (Vetter et al., 1999), but have intact outer and inner hair cells, intact but morphologically and functionally abnormal efferent synapses, normal cochlear nucleus innervation by MOC axon collaterals, and normal hearing thresholds in young adulthood (May et al., 2002; Brown and Vetter, 2009; Lauer and May, 2011). The MOC dysfunction in this strain leads to auditory processing deficits that are exacerbated by exposure to elevated environmental noise levels (Lauer and May, 2011; May et al., 2011). α9KO mice also show abnormal axonal pruning and response properties in the lateral superior olive (Clause et al., 2014). In the present study, we used this mouse model to test the hypotheses that mice with dysfunctional MOC activity would show accelerated onset of hearing loss with age, and that the hearing loss would be further accelerated by exposure to elevated environmental noise levels that challenge the MOC system.

## Materials and methods

### Subjects

Fifty-five adult male and female homozygous α9KO (CBACaJ;129S-*Chrna9*^*tm*1*Bedv*^/J, strain #005696) and from our breeding colony originally established from founders obtained from the Jackson Laboratory cryopreservation store (α9KO) 47 homozygous wild type (WT) mice generated as F1 CBA/CaJ and 129/SvEvTac were used in the study. Homozygous breeding pairs from our breeding colony were randomly selected for housing in filter top shoebox cages quiet or noisy animal housing areas to ensure an adequate number of animals of each genotype (Lauer et al., 2009). Background sound levels were below 40 dB APL in 1/3 octave bands with frequencies between 1 and 20 kHz in the quiet room, whereas sound levels were 40–45 dB SPL in much of this range due to cage ventilation systems in this room. The largest differences in sound levels occurred below 10 kHz. Very little ultrasonic sound existed in the room. Additional transient sounds that exceeded the background by 20–30 dB were generated during the day by experimenters and animal care staff, and activity was more frequent in the noisy room due to high-density housing. After weaning, mice were housed in same-sex groups of 3–5. Mice has access to *ad libitum* food and water and were kept on a 12:12 h light cycle in rooms maintained at 70–79°F and 30–50% humidity. Animals were tested up to 15 months of age. The α9KO mice frequently developed dermatitis at mid-to-older ages, requiring treatment with ototoxic substances (not conducive to further hearing studies) or euthanasia. Thus, animals could not be tested in large numbers at older ages as the sample was limited to those not treated with antibiotics or euthanized for tissue harvest. In addition, we observed that the strain was resistant to anesthesia and developed rapid tolerance with repeated testing. Though some animals were tested repeatedly, some could only be tested at one age. The four main subject groups are denoted as follows: wild type housed in the quiet room (WT-Q), α9KO housed in the quiet room (α9KO-Q), wild type housed in the noisy room (WT-N), α9KO housed in the noisy room (α9KO-N). Sound levels in the room were periodically monitored using a data-logging sound level meter with 1/3 octave band measurement capabilities (Larson-Davis LxT2) and were detailed in our previous studies (Lauer et al., 2009; Lauer and May, 2011). All procedures were approved and performed in accordance with the Guide for the Care and Use of Laboratory Animals and the Johns Hopkins University Animal Care and Use Committee.

### Auditory brainstem response (ABR) recordings and analysis

Recording procedures were similar to those previously described (Lauer and May, 2011; Lina and Lauer, 2013; McGuire et al., 2015). Mice were anesthetized with 100 mg/kg ketamine and 20 mg/kg xylazine (i. p.) and placed on an electronically controlled heating pad inside a small sound-attenuating chamber 30 cm from a pair of Radio Shack Supertweeters in front of the animal. The animals' core temperature was maintained at 37°C ± 1°. ABRs were differentially recorded from the scalp using subcutaneous platinum needle electrodes (G.R.A.S.) placed over the left bulla and at the vertex of the skull, with a ground electrode inserted into the leg muscle. Responses were amplified (ISO-80, World Precision Instruments) and bandpass filtered from 30 to 3,000 Hz (Krohn-Hite). Stimulus generation and ABR measurements were controlled and collected using Tucker Davis technologies programming modules, custom Matlab-based software, and a PC. Responses were averaged over 300 stimulus presentations. Stimuli were clicks and 5-ms tone pips (8, 16, 32 kHz) with a rise/fall time of 0.5 ms played at a rate of 10/s. Stimuli were calibrated with a ¼″ Bruel & Kjaer microphone placed at the location normally occupied by the mouse's head during testing using a custom Mathworks Matlab-based program.

**Figure 1:**
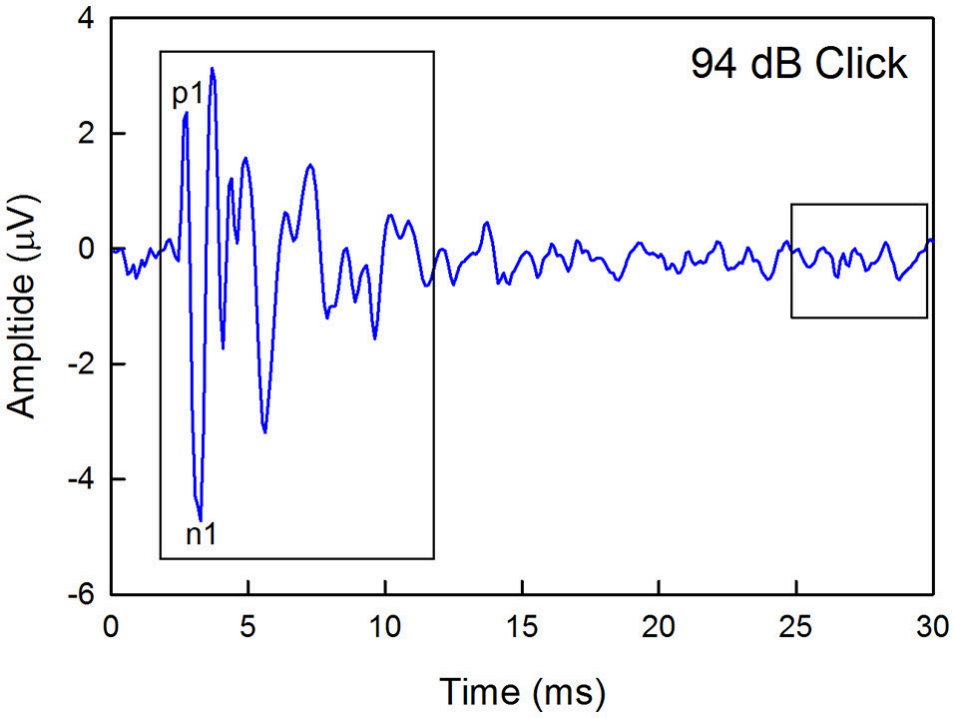
Example of a click-evoked ABR recorded from a wild type mouse housed in quiet. Thresholds were estimated by recording responses to a descending level series of stimuli, calculating the maximum peak-to-peak amplitude (any peak) within a 10-ms window starting at 2-ms after the onset of the stimuli, and determining the sound level that produced a response that was 2 standard deviations about the average baseline noise recorded in a 5-ms window at the end of each recording epoch. The waveform latency is not corrected for the travel time between the speaker and the ear. The amplitude of the first positive (p1) to the first negative (n1) peaks were also calculated.

Thresholds and suprathreshold responses were measured by presenting a descending series of stimulus levels beginning with −10 dB of the maximum possible output of the speaker and continuing in 5 or 10 dB steps until no response could be discerned from the noise. Threshold was defined as the sound level at which the ABR amplitude (any wave) was 2 standard deviations above the average level of a 5 ms window of baseline noise collected at the end of a 30-ms recording epoch (Figure 1). Responses to clicks were measured first to ensure an optimal signal-to-noise ratio and the presence of at least four distinct peaks. Responses to tones were measured in a pseudorandom order. A second method of testing age-related hearing loss was not used because operant conditioning procedures require months of training and testing for each frequency, precluding repeated screening across frequency in three age groups.

Previous studies have linked auditory nerve damage to reduced ABR wave 1 amplitude in the absence of threshold shifts (Sergeyenko et al., 2013; Kujawa and Liberman, 2015). Thus, wave 1 amplitudes for the highest level stimuli were measured manually offline for the 1–5 month and 11–15 month old groups by marking the positive and negative peaks and measuring the difference between each positive peak and the immediately following negative deflection as demarcated in Figure 1. Sound levels were 94 dB for clicks, 101 dB for 8 kHz, 99 dB for 16 kHz, and 76 dB for 32 kHz.

### Statistical analysis

Nonparametric statistical tests were performed on the data because some of the distributions were non-normal, the sample sizes were not equal across all groups, and these tests are more robust because they rely on fewer assumptions than parametric statistics. We performed Kruskal-Wallis rank sum tests to identify significant main effects of group at a particular age and frequency, or effects of age within a particular group using a criterion of *p* < 0.05. If the main effect was significant, *post-hoc* Mann-Whitney *U*-tests were performed with a *P*-value adjusted for multiple comparisons by dividing *p* = 0.05 by the number of pairwise comparisons performed. *Post-hoc* comparisons were performed on the following group pairs: WT-Q v. α9KO-Q, WT-N v. α9KO-N, WT-Q v. WT-N, α9KO-Q v. α9KO-N, with an adjusted significance criterion of *p* < 0.0125. *P*-values of < 0.001 could not be computed by the statistics software and are therefore reported as *p* < 0.001. Data distributions are represented as box plots (Krzywinski and Altman, 2014), and the median is depicted by a line. Dots represent the 5th and 95th percentiles.

## Results

### ABR thresholds

**Figure 2:**
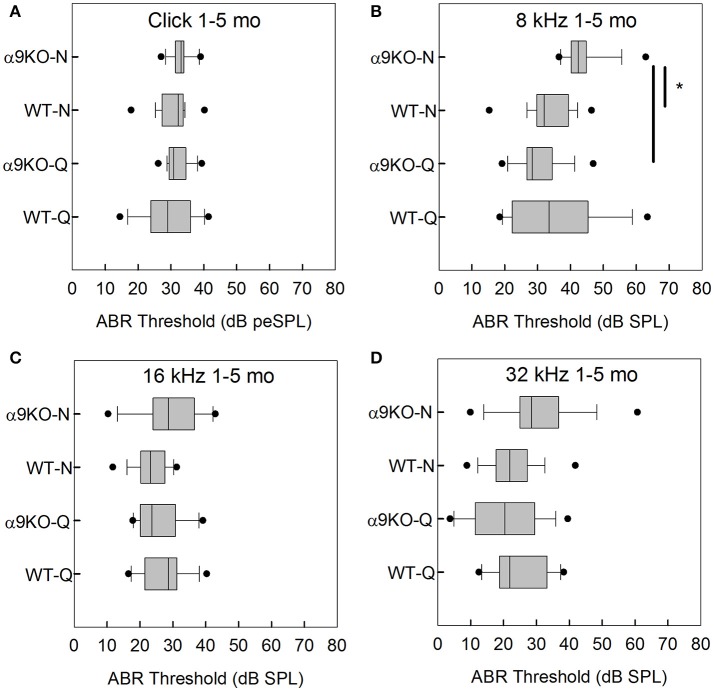
Distribution of ABR click and tone thresholds in four groups of 1–5 month old mice: wild type mice housed in quiet (WT-Q), wild type mice housed in noisy conditions (WT-N), olivocochlear-deficient alpha9 nicotinic acetylcholine receptor knockout mice housed in quiet (α9KO-Q), alpha9 nicotinic acetylcholine receptor knockout mice housed in noisy conditions (α9KO-N). Horizontal lines inside the boxplots represent the median. Dots represent the 5th and 95th percentiles. Thick black lines and asterisks indicate statistically significant differences identified with *post-hoc* analysis. Thresholds are shown for click **(A)**, 8 kHz **(B)**, 16 kHz **(C)**, and 32 kHz **(D)**.

The distributions of ABR thresholds measured for four frequencies in 1–5 month old mice from each of the four groups (*n* = 14 WT-Q, *n* = 20 α9KO-Q, *n* = 20 WT-N, *n* = 15 α9KO-N) are shown in Figure 2. There was no significant main effect of group for clicks (*H* = 5.238, *p* = 0.155), 16 kHz (*H* = 5.754, *p* = 0.124), and 32 kHz (*H* = 5.703, *p* = 0.127). There was a significant main effect of group for 8 kHz (*H* = 28.174, *p* < 0.001). *Post-hoc* comparisons revealed significant differences between the 8 kHz thresholds of α9KO-Q and α9KO-N (*p* < 0.001) and α9KO-N and WT-N (*p* < 0.001). Thresholds were higher in α9KO-N than WT-N and α9KO-Q for 8 kHz.

**Figure 3:**
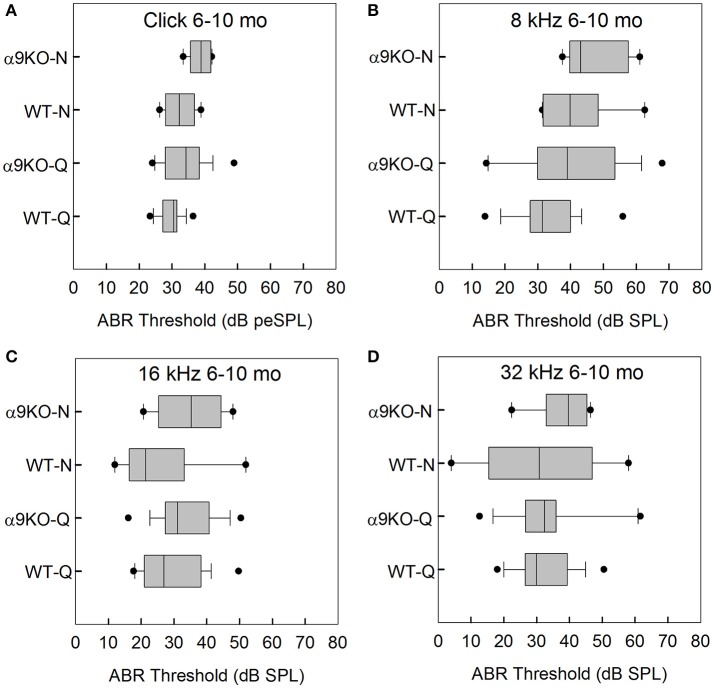
Distribution of ABR click and tone thresholds in four groups of 6–10 month old mice: wild type mice housed in quiet (WT-Q), wild type mice housed in noisy conditions (WT-N), olivocochlear-deficient alpha9 nicotinic acetylcholine receptor knockout mice housed in quiet (α9KO-Q), alpha9 nicotinic acetylcholine receptor knockout mice housed in noisy conditions (α9KO-N). Horizontal lines inside the boxplots represent the median. Dots represent the 5th and 95th percentiles. Thresholds are shown for click **(A)**, 8 kHz **(B)**, 16 kHz **(C)**, and 32 kHz **(D)**.

The distributions of ABR thresholds measured for four frequencies in 6–10 month old mice from each of the four groups (*n* = 19 WT-Q, *n* = 16 α9KO-Q, *n* = 8 WT-N, *n* = 6 α9KO-N) are shown in Figure 3. There were significant main effects of group for click (*H* = 12.569, *p* = 0.006) and 8 kHz (*H* = 9.202, *p* = 0.027). Main effects were not significant for 16 kHz (*H* = 6.671, *p* = 0.083) and 32 kHz (*H* = 0.934, *p* = 0.817). *Post-hoc* comparisons did not reveal any significant differences for any of the pairwise comparisons tested. The significant main effects were likely driven by differences between WT-Q and α9KO-N, but these group comparisons were not of interest in the present study.

**Figure 4:**
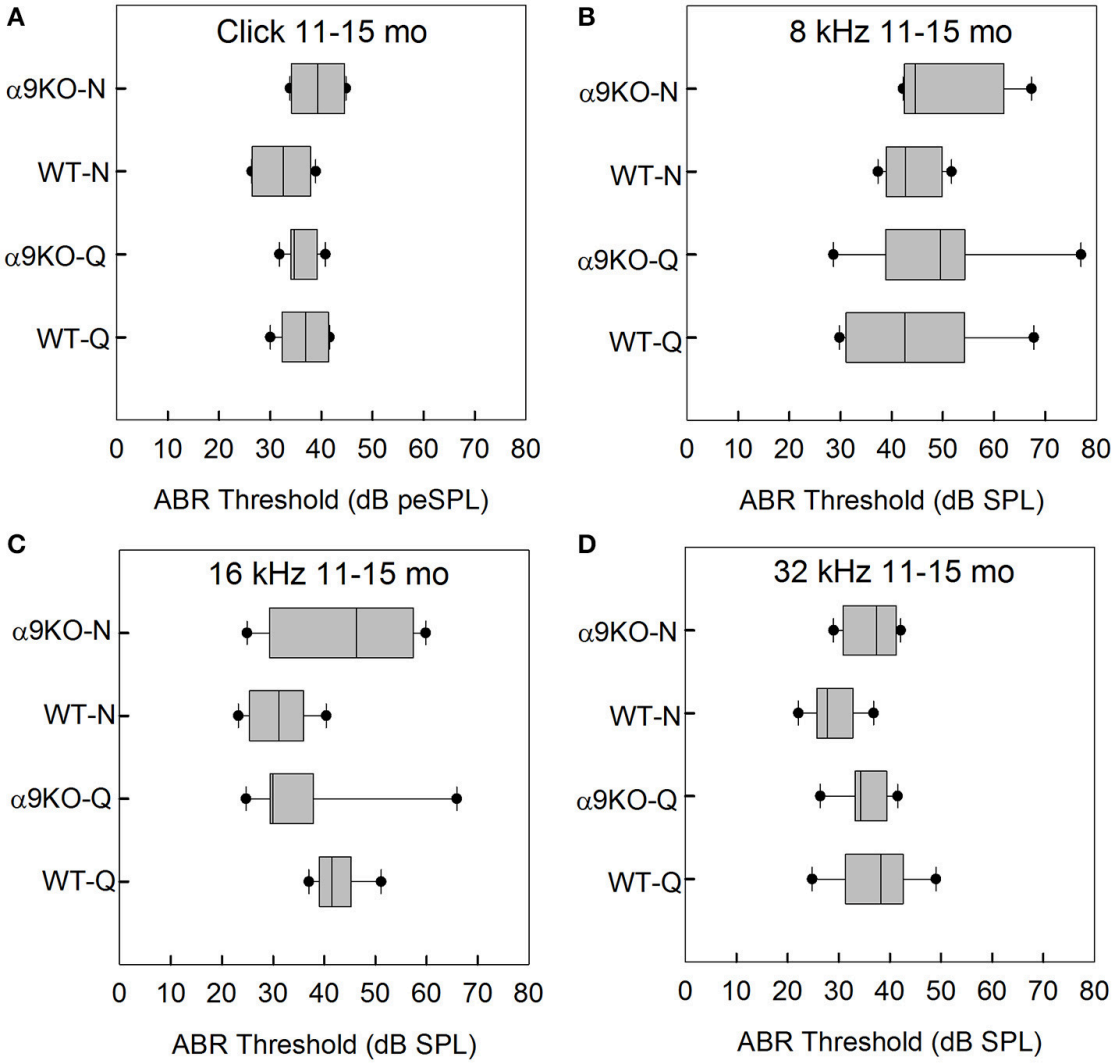
Distribution of ABR click and tone thresholds in four groups of 11–15 month old mice: wild type mice housed in quiet (WT-Q), wild type mice housed in noisy conditions (WT-N), olivocochlear-deficient alpha9 nicotinic acetylcholine receptor knockout mice housed in quiet (α9KO-Q), alpha9 nicotinic acetylcholine receptor knockout mice housed in noisy conditions (α9KO-N). Horizontal lines inside the boxplots represent the median. Dots represent the 5th and 95th percentiles. Thresholds are shown for click **(A)**, 8 kHz **(B)**, 16 kHz **(C)**, and 32 kHz **(D)**.

The distributions of ABR thresholds measured for four frequencies in 11–15 month old mice from each of the four groups (*n* = 6 WT-Q, *n* = 7 α9KO-Q, *n* = 6 WT-N, *n* = 4 α9KO-N) are shown in Figure 4. Main effects were not significant for click (*H* = 4.262, *p* = 0.234), 8 kHz (*H* = 1.376, *p* = 0.711), 16 kHz (*H* = 7.052, *p* = 0.07) and 32 kHz (*H* = 5.967, *p* = 0.113). *Post-hoc* analyses were not performed due to the non-significant main effects. Due to the small sample sizes that we were able to obtain because of complications from dermatitis in the older α9KO mice and the lack of a statistically significant effect, we performed a sample size calculation that indicated a sample size of 23 animals per group would have been required to detect a minimum 5 dB threshold difference with a power of 0.8, alpha = 0.05. Obtaining this number of 11–15 month old animals from each group would have required using animals that had to be treated with potentially ototoxic antibiotics to treat their dermatitis. The use of potentially ototoxic antibiotics could have confounded the results; therefore, the small samples size was the preferable option.

### ABR wave amplitudes

**Figure 5:**
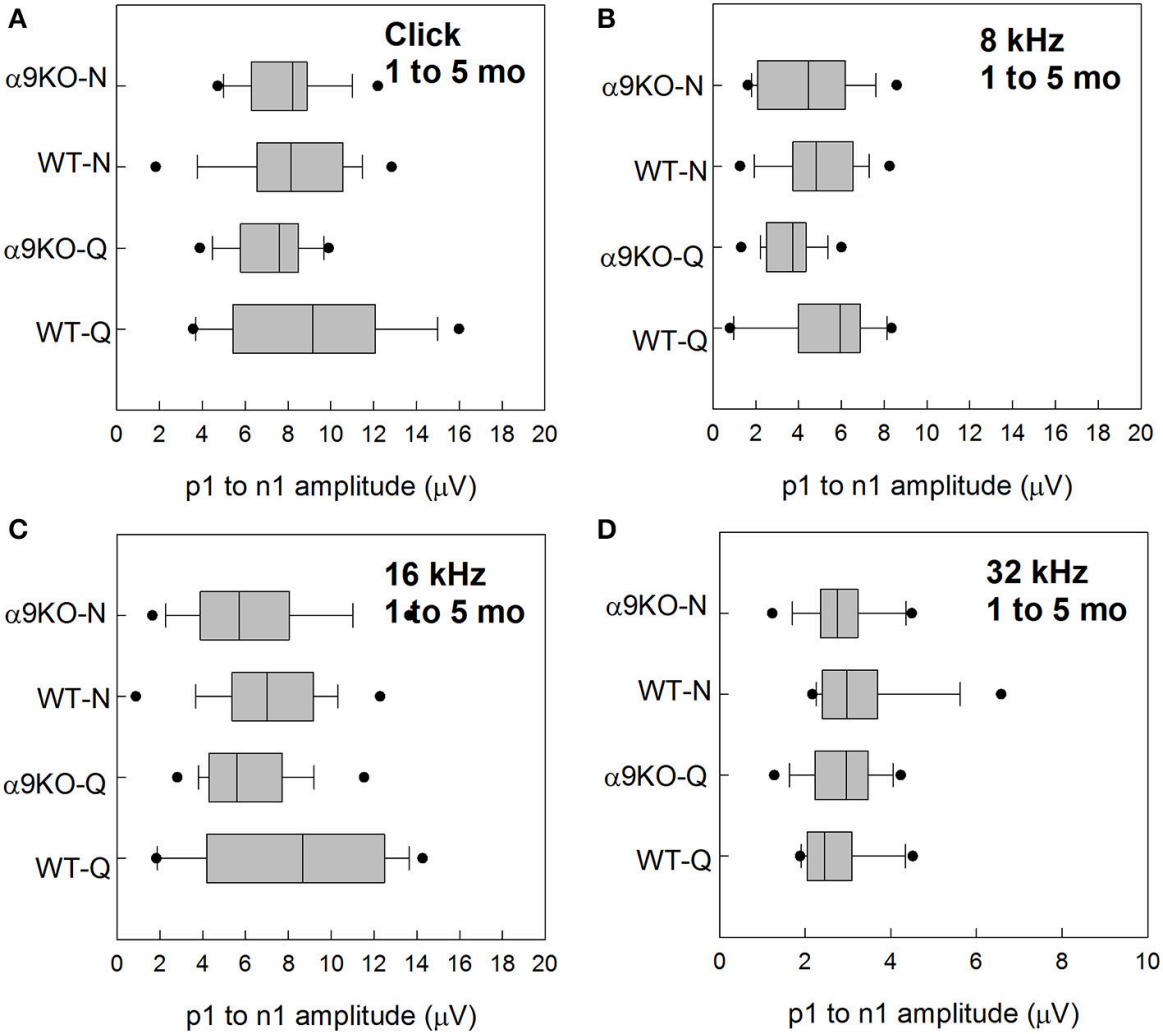
Distribution of ABR wave 1 amplitudes for clicks and tones in four groups of 1–5 month old mice: wild type mice housed in quiet (WT-Q), wild type mice housed in noisy conditions (WT-N), olivocochlear-deficient alpha9 nicotinic acetylcholine receptor knockout mice housed in quiet (α9KO-Q), alpha9 nicotinic acetylcholine receptor knockout mice housed in noisy conditions (α9KO-N). Horizontal lines inside the boxplots represent the median. Dots represent the 5th and 95th percentiles. Wave amplitudes are shown for click **(A)**, 8 kHz **(B)**, 16 kHz **(C)**, and 32 kHz **(D)**.

**Figure 6:**
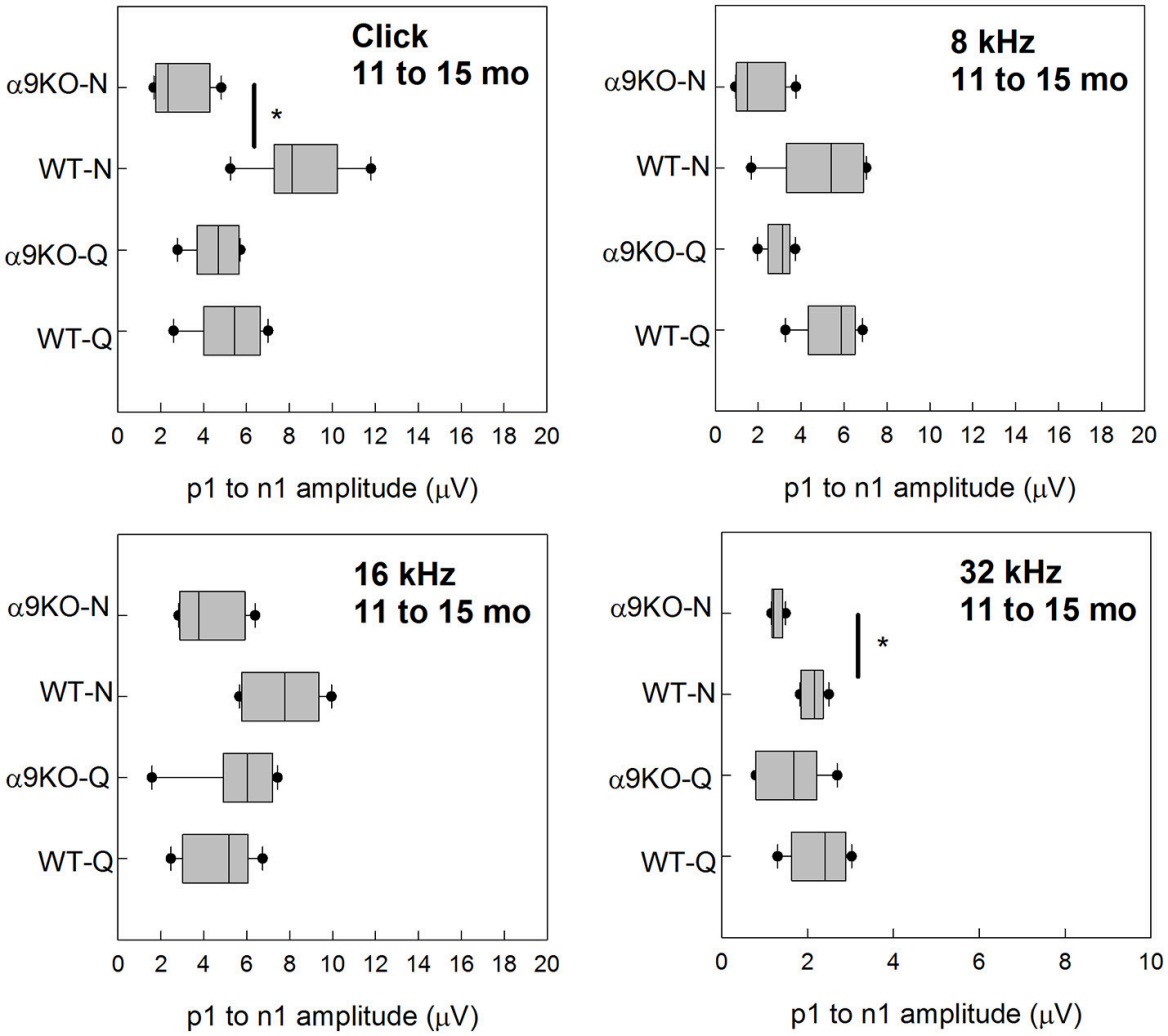
Distribution of ABR wave 1 amplitudes for clicks and tones in four groups of 11–15 month old mice: wild type mice housed in quiet (WT-Q), wild type mice housed in noisy conditions (WT-N), olivocochlear-deficient alpha9 nicotinic acetylcholine receptor knockout mice housed in quiet (α9KO-Q), alpha9 nicotinic acetylcholine receptor knockout mice housed in noisy conditions (α9KO-N). Horizontal lines inside the boxplots represent the median. Dots represent the 5th and 95th percentiles. Thick black lines and asterisks indicate statistically significant differences identified with *post-hoc* analysis. Wave amplitudes are shown for click **(A)**, 8 kHz **(B)**, 16 kHz **(C)**, and 32 kHz **(D)**.

Wave 1 amplitudes for 1–5 month old mice were similar across groups as shown in Figure 5. No significant effects were observed for any frequency: click (*H* = 3.82, *p* = 0.282), 8 kHz (*H* = 7.527, *p* = 0.057), 16 kHz (*H* = 4.78, *p* = 0.189), and 32 kHz (*H* = 1.963, *p* = 0.58). Wave 1 amplitudes for 11–15 month old mice are shown in Figure 6. Wave amplitudes were not smaller in α9KO mice than in WTs, but WT-N mice tended to have larger wave 1 amplitudes than the other groups. Wave 1 amplitudes were not significantly different between α9KO-Q and WT-Q, between WT-Q and WT-N, or between α9KO-Q and α9KO-N. However, wave 1 was significantly different between WT-N and α9KO-N for clicks (*p* = 0.01) and 32 kHz (*p* = 0.01).

## Discussion

Several studies in animals and humans have hypothesized that weakened MOC feedback results in accelerated hearing loss with age and chronic environmental noise exposure. We tested for hearing loss by measuring ABRs at a range of ages and with exposure to quiet and noisy environmental in α9KO mice (Vetter et al., 1999). Our results did not find accelerated onset of hearing loss in α9KO mice compared to WT mice up to 15 months of age. All groups showed ~5–15 dB ABR threshold increases with age, but the α9KO mice did not show larger threshold increases than WTs. Increased environmental noise exposure also did not result in substantially accelerated hearing loss in α9KO mice. Young adult α9KO mice housed in a high-traffic, noisy vivarium showed mild threshold impairments at 8 kHz, but this difference was not maintained at later ages. ABR wave 1 amplitude measurements also did not show accelerated impairments with age in α9KO mice housed in quiet. Housing in a noisy environment resulted in smaller ABR wave 1 amplitudes in older α9KO mice compared to WTs housed in noise, but not between α9KO housed in quiet and noise. This difference was primarily due to the unusually large wave 1 amplitudes in WTs housed in noise, even compared to WTs housed in quiet, possibly due to upregulation of efferent protective mechanisms by noise in this group. In aggregate, the present results suggest that impaired cholinergic suppression of outer hair cell activity by the medial OC efferent synapse does not result in early onset of hearing loss in α9KO mice.

The present results from α9KO mice are in contrast to what has been reported following OC lesions in mice (Maison et al., 2013; Liberman et al., 2014), as well as some of the contralateral suppression studies in mice and humans linking increased thresholds with reduced or absent MOC feedback (Parthasarathy, 2001; Kim et al., 2002; Jacobson et al., 2003; Varghese et al., 2005; Zhu et al., 2007). De-efferentation increases loss of auditory nerve ribbon synapses with age, and loss of MOC fibers most strongly affects mid to low frequencies (Liberman et al., 2014). These observations were made at ages corresponding to our older age group (~13 months). Functional discrepancies between lesioned animals and α9KO mice have also been shown in behavioral studies. Lesioned animals show hearing in noise deficits under certain stimulus conditions, whereas α9KO mice show normal hearing in noise capabilities (Dewson, 1968; Trahiotis and Elliott, 1970; Igarashi et al., 1972, 1977, 1979a,b; Hienz et al., 1998; May et al., 2002, 2004). An additional factor to consider is that most previous sound-driven assays of medial olivocochlear strength are now thought to be dominated by the middle ear muscle reflex or combined pathways in mice and rabbits (Whitehead et al., 1991; Chambers et al., 2012; Maison et al., 2012; Xu et al., 2017), thereby calling into question the role of the olivocochlear system in many previous studies of hearing protection. A limitation of the present study is the smaller sample size of α9KO mice tested at 11–15 months. It remains possible that differences in age-related hearing loss could emerge at older ages in α9KO mice, or with different noise exposure conditions, but health concerns precluded testing animals at ages older than 15 months or a larger sample size of animals in the 11–15 month age group. However, a trend toward increased thresholds relative to WT mice was not observed. Future studies may circumvent this issue by using a different background strain or more intense noise exposures. It is possible that background strains that are more susceptible to age-related or noise-induced hearing loss may reveal interesting interactions with the deficient olivocochlear activity.

Loss of MOC synapses from the outer hair cells may accelerate hearing loss due to loss of trophic factors. Several studies in mutant mouse strains have associated loss of MOC synapses from outer hair cells with accelerated age-related hearing loss. A transgenic reporter mouse strain generated on the C57BL/6J background, a strain with early onset hearing loss (Mikaelian, 1979; Hequembourg and Liberman, 2001; Prosen et al., 2003), shows a loss of YFP-positive MOC synapse labeling at 12 months of age (Fu et al., 2010). At this age, these mice show hearing loss of about 30–45 dB at all frequencies compared to 2 month old mice of the same strain, with only scattered outer hair cell loss. Heterozygous mice with an IsL1 transgene also show accelerated age-related hearing loss, partial outer hair cell loss, and partial MOC terminal loss (Chumak et al., 2016). These mice exhibit a number of other abnormalities, including hyperactivity, circling behavior, and a reduced complement of spiral ganglion neurons, so it is difficult to tease apart the various central and peripheral factors contributing to loss of MOC terminals and hearing loss in this strain. Thymine-deficient mice also show hearing loss corresponding to some loss of MOC terminals and ribbon synapses, particularly in the apex (Maison et al., 2016).

In all of these studies, it is difficult to know if the hearing loss results from MOC terminal loss or if the terminal loss occurs in response to the hearing loss. Smaller alpha-synuclein and synaptophysin labeled terminals have been observed in the mid and apical cochlear regions in young adult C57 mice compared to CBA mice (mice obtained in Korea, full strain information not specified), suggesting that the C57 strains may have pre-existing MOC deficiencies (Park et al., 2011). Whether or not this is directly related to the early age-related hearing loss cadherin23 mutation (Di Palma et al., 2001), or is triggered by early ribbon synapse loss (Stamataki et al., 2006) in this strain is unknown. Aging C57BL6 mice also experience an increase in inner hair cell efferent innervation that inhibits hair cell activity (Lauer et al., 2012; Zachary and Fuchs, 2015).

The data from mutant mouse strains suggests that hearing loss resulting from MOC synapse loss is possible, but it is not necessarily a characteristic of accelerated onset of age-related hearing loss and may involve other forms of OC reorganization. At present it is unclear how MOC or LOC synapses change with age in humans or other strains of mice. Substantial reorganization of efferent synapses may serve to minimize excitotoxic damage to auditory nerve dendrites, which could prevent accumulated damage from everyday noise exposures (Nouvian et al., 2015).

## Conclusions

The results of the present study do not support the hypothesis that weakened MOC feedback contributes to early onset age-related hearing loss. Much remains to be explored regarding the compensatory and protective capacity for plasticity in the adult OC system. It remains possible that deficient MOC activity may result in age-related suprathreshold perceptual deficits, especially in noise. The largest effects of MOC feedback are reported to occur on low spontaneous rate, high threshold auditory nerve fibers (Guinan and Stankovic, 1996). These fibers are especially vulnerable to acoustic overexposure (Furman et al., 2013). Damage to these fibers may manifest as deficient spectral representation of sounds in noise, which could be further exacerbated by deficient MOC noise suppression (Reiss et al., 2011). Consistent with this hypothesis, behaviorally trained α9KO mice showed some age-related changes in intensity discrimination and tone detection in noise around 8–12 months of age, but the small sample size dictated by the labor-intensive nature of operant conditioning studies may have obscured the magnitude of the changes (May et al., 2002). Future studies of the role of MOC feedback in age-related hearing decline should consider more complex stimulus paradigms to identify deficits that are not reflected in conventional tone- or click-evoked ABR measures.

## Author contributions

AL: designed and performed the experiments, analyzed the data, and wrote the manuscript.

### Conflict of interest statement

The author declares that the research was conducted in the absence of any commercial or financial relationships that could be construed as a potential conflict of interest.
